# Effect of osteogenesis imperfecta on children and their families

**DOI:** 10.1186/1687-9856-2013-S1-P169

**Published:** 2013-10-03

**Authors:** Vu Chi Dung, Kate Armstrong, Can Thi Bich Ngoc, Bui Phuong Thao, Nguyen Ngoc Khanh, Nguyen Thu Trang, Nguyen Thi Hoan, Nguyen Phu Dat, Craig Munns

**Affiliations:** 1 Vietnam National Hospital of Pediatrics. Hanoi, Vietnam; 2 CLAN (Caring & Living As Neighbours; 3 Children’s Hospital at Westmead, University of Sydney, Sydney, Australia

## 

Osteogenesis Imperfecta (OI) is a heterogeneous genetic disorder, with features that include increased bone fragility, pathological fractures, blue sclera, dentinogenesis imperfecta and conductive or mixed hearing loss. Clinical variability is wide from children with few fractures and normal stature to children with multiple fractures, long bone deformity, scoliosis and extreme short stature. Although there is no curative treatment, there are several therapeutic tools capable of improving the course of the condition and patient quality of life.

We aim to evaluate the effect of OI on the well being of children with the disorder and their families through a family-centered questionnaire. Sixty children with OI from the Vietnam National Hospital of Pediatrics and/or their parent(s), who attended the first annual family support group in 2011, completed a child and parent questionnaire.

60 patients participated, 26 female and 34 male. The median age was 6.0 years [interquartile range (IQR) 0.25 - 18 years]. Of these, 36 (60%) had dentinogenesis imperfect and 23 (38.3%) had a scoliosis. The median number of fractures was 6.0 (IQR 0 – 30) and median number of hospitalizations due to OI was 5.0 (IQR 0-30). Among patients of school age, 9 (15%) could not go to school due to OI. Almost all parents (93.7%) worried about school social communication of the patients. Among these parents, 100% fear of inferiority with friends and 98.3% fear of broken bones. Most parents (76.2%) were significantly concerned about their child's health. The parents’ themselves reported psychological concerns, with feelings of desperation (58.4%), anxiety (81.7%) and depression (56.7%).

Osteogenesis imperfecta appeared to have a significant deleterious effects on the life of the patients and their families. These data provide a baseline from which to evaluate the effectiveness of interventions to improve the medical and psychological needs of this cohort and their families.

